# Case Report: Expanding the tumour spectrum associated with the Birt-Hogg-Dubé cancer susceptibility syndrome

**DOI:** 10.12688/f1000research.4205.1

**Published:** 2014-07-11

**Authors:** Patrick R. Benusiglio, Sophie Gad, Christophe Massard, Edith Carton, Elisabeth Longchampt, Tiffany Faudot, Jérôme Lamoril, Sophie Ferlicot

**Affiliations:** 1 Centre Expert National Cancers Rares PREDIR, Hôpital Bicêtre, AP-HP, Le Kremlin Bicêtre, 94275, France; 2 Département de Médecine Oncologique, Gustave Roussy Cancer Campus, Villejuif, 94805, France; 3 Laboratoire de Génétique Oncologique EPHE, INSERM U753, Faculté de Médecine Paris-Sud, Le Kremlin-Bicêtre et Gustave Roussy Cancer Campus, Villejuif, 94276, France; 4 Service d’Anatomie Pathologique, Hôpital Foch, Suresnes, 92151, France; 5 Département de Génétique Moléculaire, Hôpital Bichat – Claude Bernard, AP-HP, Paris, 75018, France; 6 Service d’Anatomie Pathologique, Hôpital Bicêtre, AP-HP, Le Kremlin-Bicêtre, 94275, France

## Abstract

Patients with the Birt-Hogg-Dubé cancer susceptibility syndrome are at high risk of developing renal cell carcinoma, pulmonary cysts and pneumothorax, and skin lesions called fibrofolliculomas. Here we report the case of a Birt-Hogg-Dubé patient with a primary clear cell carcinoma of the thyroid (a very rare type of thyroid cancer), and *FLCN *loss of heterozygosity within the tumour, providing molecular evidence for this association. Our findings expand the tumour spectrum associated with this syndrome. It is paramount to identify individuals with Birt-Hogg-Dubé so that they, and subsequently their affected relatives, can benefit from tailored cancer screening and prevention.

Birt-Hogg-Dubé (BHD) is a cancer susceptibility syndrome caused by dominantly-inherited mutations in the folliculin gene *FLCN*. Affected individuals are at risk of renal cell carcinoma (RCC), spontaneous pneumothorax associated with lung cysts and white skin papules called fibrofolliculomas ^1^. RCC affects 34% of mutation carriers ^2^, and most tumours are of chromophobe, oncocytoma, hybrid or clear cell histology ^3^. Thirty-eight and 84% of BHD cases have a history of pneumothorax and fibrofolliculomas, respectively ^2^. We report the case of a BHD patient with a primary clear cell carcinoma of the thyroid and provide molecular evidence supporting an association between the BHD syndrome and this rare tumour.

## Case report

**Figure 1.**
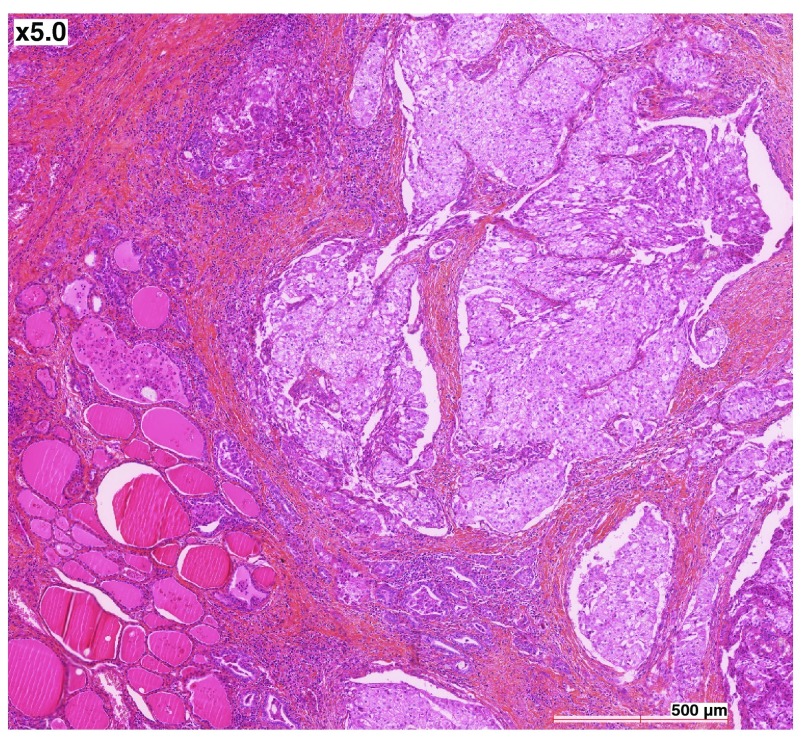
Primary clear cell carcinoma of the thyroid. The neoplastic cells have a small, peripheral nucleus, with abundant clear cytoplasm. Nests of carcinomatous elements are separated by a fibrous vascularized stroma. Hematoxylin and eosin staining, 5×.

**Figure 2.**
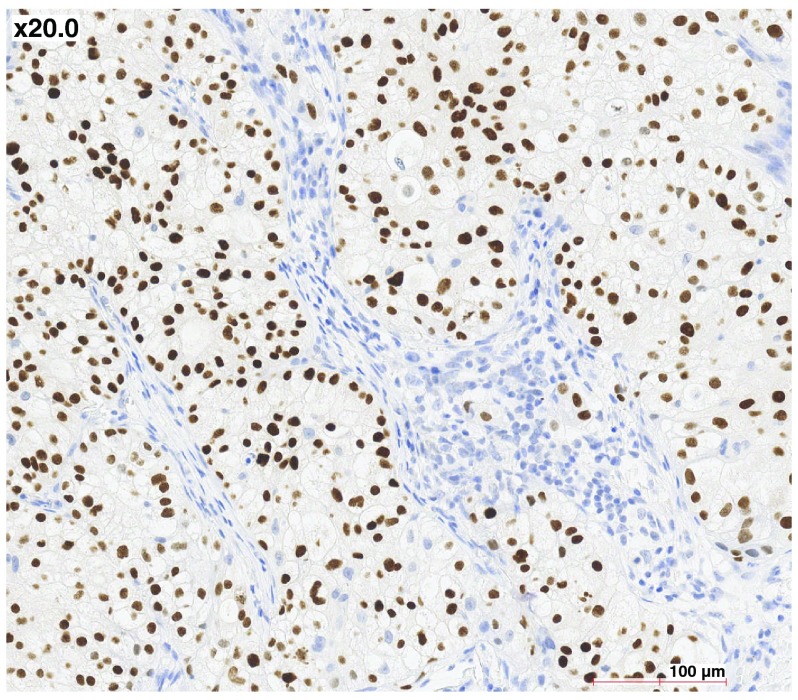
Lung metastases. Nuclear staining for TTF1 is strongly positive, 20×.

A 72 year-old French Caucasian male with a history of hypertension and early-stage rectal carcinoma (diagnosed at the age of 64) complained of a right thyroid nodule. Thyroid function tests were normal. Medication at the time consisted of irbesartan, lercanidipine and aspirin. The nodule was suspect on echography, and an operation was scheduled. Intraoperative fresh frozen analysis confirmed the malignant nature of the lesion, and thyroidectomy with paratracheal lymph node dissection was performed. The pathologist described a poorly circumscribed tumour measuring approximately 3.5 × 2 cm. On microscopic examination, nests of carcinomatous elements separated by a fibrous vascularized stroma were observed ( Figure 1, hematoxylin and eosin). The neoplastic cells had a small, peripheral nucleus, with abundant clear cytoplasm. There was limited invasion of the capillaries, but widespread infiltration of the surrounding normal thyroid parenchyma and of the surgical margins. Staining was positive for cytokeratin 7 (Dako OV-TL 12/30), and negative for cytokeratin 20 (Dako Ks 20.8) and thyroglobulin (Dako DAK-Tg6). Lymph nodes were free of tumours. Histological examination was suggestive of a clear cell RCC metastatic to the thyroid, but no primary lesion was seen on 18-F fluorodeoxyglucose (FDG) positron emission tomography/computerised tomography (PET/CT) scans, in the kidneys or elsewhere. It was therefore concluded that the patient had a primary clear cell carcinoma of the thyroid. In the absence of papillary structures, significant nuclear grooves or pseudoinclusions, the tumour was considered a variant form of follicular carcinoma. Adjuvant radiotherapy was administered (46 Gy over five weeks). Two years later, multiple bilateral pulmonary nodules were seen on follow-up PET/CT scan. Two were surgically resected, and their microscopic appearance matched what had been observed in the thyroid two years earlier, except that complementary analyses now showed positive nuclear staining for Thyroid Transcription Factor 1 (TTF-1) ( Figure 2), confirming as a result that the organ primarily affected was indeed the thyroid. There were no metastases in other organs, and the kidneys, as seen previously, were free of tumour.

The patient also had multiple pulmonary air-filled cysts on baseline and follow up PET/CTs, as well as a right recurring pneumothorax. Family history was relevant as his son and two nephews had a history of spontaneous pneumothorax. On dermatological examination, one could see face fibrofolliculomas. Both these pulmonary and dermatological features were highly suggestive of BHD, and a blood sample was sent for *FLCN* analysis. Sequencing of the exons and of their flanking regions was performed with the Big Dye Terminator v.1.1 kit on the ABI 3730 sequencer (Applied Biosystems), and a search for large deletions was done using Multiplex Ligation-dependent Probe Amplification (MLPA, MRC-Holland). The c.1062G>C mutation in exon 9, which is classified as pathogenic by the SIFT ( http://sift.jcvi.org/), Polyphen ( http://genetics.bwh.harvard.edu/pph2/) and SNPs3D ( http://www.snps3d.org/) bioinformatics prediction tools, was identified. It likely interferes with intron 10 splicing as it is located on the last base of exon 9, might affect a key splice site ( http://www.umd.be/HSF/, http://genes.mit.edu/burgelab/maxent/Xmaxentscan_scoreseq.html), and is adjacent to the already known c.1062+1G>A splicing mutation ( https://grenada.lumc.nl/LOVD2/shared1/home.php). The diagnosis of BHD was therefore confirmed.

**Figure 3.**
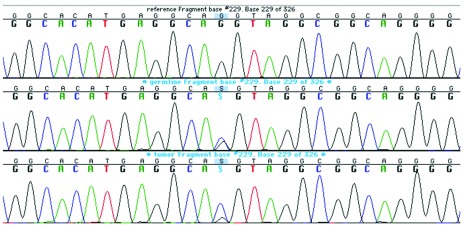
*FLCN* sequences. The c.1062G>C wild type allele in exon 9 is lost in the tumour, while the patient is heterozygote for the mutation in the germline. Top sequence, control DNA. Middle sequence, germline DNA (normal tissue). Bottom sequence, tumour DNA.

To investigate the association between BHD and the thyroid carcinoma, we performed *FLCN* analysis on tumoral and adjacent normal tissues. DNA was extracted from frozen sections with the QIAamp DNA mini kit (Qiagen), and exons and exon-intron junctions were sequenced with the methods decribed above. The c.1062G>C wild type allele was lost in the tumour, strongly suggesting loss of heterozygosity (LOH) ( Figure 3).

Two years after the identification of the pulmonary metastases, the patient remains clinically well. His WHO performance status is 1. He has been included in a clinical trial at the Gustave Roussy Cancer Campus, and is now on temsirolimus and cetuximab. Previous lines of treatment with gemcitabine-oxaliplatine and docetaxel have had little effect on the tumour.

## Discussion

Primary clear cell carcinoma of the thyroid is very rare. In two retrospective studies previously published, only three and four of 2784 and 572 thyroidectomies respectively were primary clear cell carcinomas ^4, 5^. Such a diagnosis is made when at least 75% of the tumour cells show marked cytoplasmic clearing ^6^. This morphological pattern can occur in nearly all major thyroid tumour types, and is observed with the accumulation of vesicles derived from mitochondria, glycogen, lipid droplets, thyroglobulin or mucin ^7^.

To our knowledge, this is the first time that the association between a non-renal clear cell carcinoma and BHD has been demonstrated. *FLCN* is a tumour suppressor gene, and associated tumours arise when both copies are inactivated most often through “second hit” somatic mutations ^8^. In our patient, LOH provides molecular evidence for the inactivation of the second copy of *FLCN*. Interestingly, the microscopic features of thyroid clear cell carcinoma are similar to those of clear cell RCC, a tumour typically associated with BHD. The TTF-1 positivity and the absence of renal lesions on successive PET/CTs confirmed that our patient’s carcinoma originated in the thyroid, and not in the kidneys. As for the negativity for thyroglobulin, it was not unexpected since this staining is notoriously inconsistent in clear cell tumours of the thyroid ^9^.

One should enquire about a personal or family history of BHD manifestations in patients with a diagnosis of thyroid clear cell carcinoma, and refer them for germline *FLCN* analysis when appropriate. Adult relatives of mutation carriers can then undergo targeted genetic testing. It is paramount to identify patients with BHD, as they are offered regular cancer screening with annual renal imaging (we alternate MRI and ultrasound imaging) and benefit from lifestyle recommendations. We advise patients not to smoke in order to minimize the risk of pneumothorax, and we inform them that activities such as deep sea diving or flying can trigger rupture of pulmonary cysts via changes in the atmospheric pressure, and that shortness of breath or chest pain in this context is likely due to pneumothorax ^10^.

Our report is of high scientific interest as, to our knowledge, no such case has ever been reported. In addition, we believe that it will increase awareness of BHD in the medical community, as this syndrome is too often overlooked even when obvious clinical manifestations are present.

## Consent

The patient described in this manuscript has provided informed written consent for his medical history and clinical images to appear in a scientific article.
