# Supplementary material for: Case Report: Expanding the tumour spectrum associated with the Birt-Hogg-Dubé cancer susceptibility syndrome
Source: F1000Res. 2014 Jul 11;3:159. [Version 1] doi: 10.12688/f1000research.4205.1 (PMC4168750; doi:10.12688/f1000research.4205.1)
Supplement: Peer review history

# Peer review: Referee response for version 1

**DOI:** 10.5256/f1000research.4503.r6020

**Published:** 2014-09-03

**Authors:** Andre-Pascal Sappino

**Affiliations:** 1 Centre d'oncologie et d'hématologie, Clinique des Grangettes, Chêne-Bougeries, Switzerland

The case report submitted by Benusiglio *et al.* is well documented and provides convincing evidence that we should consider widening the tumor spectrum generally ascribed to the rare Birt-Hogg-Dubé syndrome. Indeed, the authors report the case of a 72 y-old patient with a suggestive familial and personal history who developed a primary clear-cell carcinoma of the thyroid. Gene-testing for FLCN revelead a germ-line deleterious mutation and LOH for FLCN was demonstrated in the thyroid tumoral tissue. This finding is of interest since most (all ?) cancers described so far in this syndrome arise from the kidneys.

Altogether, the title, the abstract, and the article content, based on adequate methodology, are appropriate.

However, we are told that the reported patient developed at the age of 64 an early-stage rectal cancer. It would have been interesting to know more about the morphological aspects of this primary digestive tumor and if the authors could also perform on this primary the search for FLCN LOH. This information may be relevant since the median age of tumor diagnosis in this syndrome is generally considered to occur below 50 y, a point that the authors fail to discuss.

I have read this submission. I believe that I have an appropriate level of expertise to confirm that it is of an acceptable scientific standard.

# Peer review: Referee response for version 1

**DOI:** 10.5256/f1000research.4503.r5429

**Published:** 2014-08-22

**Authors:** Nicole Philip

**Affiliations:** 1 Département de Génétique Médicale et de Biologie Cellulaire, Assistance Publique - Hopitaux de Marseille, Marseille, France

This short report provides new knowledge on a very rare disorder, the Birt-Hogg-Dube syndrome. The presence of LOH in the tumor is a strong argument to conclude that the tumor is related to the syndrome.This new information can be useful for clinicians to diagnose new patients and offer appropriate surveillance.

.

I have read this submission. I believe that I have an appropriate level of expertise to confirm that it is of an acceptable scientific standard.
